# The Medical and Dental Departments of the Sou. Med. College

**Published:** 1889-05

**Authors:** 


THE MEDICAL AND DENTAL DEPARTMENTS OF THE SOUTHERN MEDICAL COLLEGE.—In this number of the Journal may be seen the advertisements of both the Medical and Dental Departments of the Southern Medical College. At a meeting of the Dental Faculty on the 25th inst., the annual election of officers for the ensuing year was held, and resulted in the choice of Dr. Crenshaw as Dean and Dr. Carpenter Secretary and Treasurer. A resolution of thanks was passed to Dr. Carpenter, the retiring Dean, for the faithful and efficient manner in which he had discharged the duties of the office for the past two years.

